# Ten Instructional Design Efforts to Help Behavior Analysts Take Up the Torch of Direct Instruction

**DOI:** 10.1007/s40617-021-00640-1

**Published:** 2021-09-07

**Authors:** Trina D. Spencer

**Affiliations:** Department of Child and Family Studies, Rightpath Research & Innovation Center, University of South Florida, 13301 Bruce B. Downs Blvd. MHC 1719, Tampa, FL 33612 USA

**Keywords:** Direct Instruction, instructional design, Generative

## Abstract

Although behavior analysts are trained in discrete trial instruction, other instructional approaches like Direct Instruction are underutilized in behavior analytic practice. Direct Instruction is a specialized technology that capitalizes on sophisticated instructional design and highly effective delivery strategies. What makes Direct Instruction so powerful is that it emphasizes the development of generative repertoires and establishes them efficiently. The purpose of this article is to introduce 10 critical instructional design efforts that behavior analysts can use in their practice, regardless of the population they serve and repertoires they build. The 10 instructional design efforts are summarized in a Direct Instruction Planning Guide. Behavior analysts can follow this sequence of design efforts and refer to the guiding questions as they develop efficient instruction for their learners. In doing so, behavior analysts can take up the torch of Direct Instruction, extend this remarkable instructional approach into their research and practice, and strengthen the behavioral technology available to behavior analytic practitioners.

Motivated by dire educational conditions, Siegfried Engelmann went to work to create a powerful instructional model known as Direct Instruction (DI; see Barbash, 2021; Kame’enui, 2021). Engelmann’s drive to ensure all children are taught effectively and efficiently led to the development of several curricula to teach reading, writing, spelling, and math in the 1960s (Engelmann, 1992). Over the years and with various coauthors, his programs underwent refinement and expansion and most are currently available through McGraw Hill SRA publishers (e.g., *Reading Mastery*, *Connecting Math Concepts*, *Language for Learning*, and *Reasoning and Writing*). Although the published DI curricula are the quickest and easiest ways to apply DI in practice, behavior analysts do not need to be limited to the commercialized programs. Fortunately for behavior analysts and their clients, DI is not only a program or curriculum; it is a model for teaching that integrates specialized design principles with effective strategies of instructional delivery (Engelmann & Carnine, 1991). In a way, DI is a technology that guides the planning and promotion of small learning increments through carefully defined and prescribed teaching behaviors. As a technology, DI is 100% transferrable and applicable to behavior analytic practice.

Behavior analysts have successfully adopted, applied, refined, and transferred the technology of discrete trial instruction (Carbone et al., 2007; Leaf et al., 2017; Patterson & Hicks, 2020), yet the technology of DI remains underutilized. Despite the enormous benefits of discrete trial instruction for learners with limited repertoires and nascent generalization skills (National Autism Center, 2015), other forms of instruction are better suited for teaching complex generative repertoires (Kohler & Malott, 2014; Ming et al., 2015; Smith, 2001). Discrete trial instruction is excellent for teaching granular discriminations and new forms of behavior (Smith, 2001). Once acquired, discrete skills can be transferred to nontraining stimulus conditions and variations of the trained responses can be shaped (Skinner, 2002). With DI, however, generativity is programmed intentionally from the beginning in a sophisticated manner. The big ideas taught in DI enable efficient learning of basic and complex skills. When the teaching of these ideas is engineered skillfully, learners become better at learning.

Although DI is sometimes associated with behavior analysis, little has been done to promote its use within our field and the majority of behavior analysis students enter their professions with little to no understanding of DI. This remarkable instructional approach should be shared far and wide. However, for DI to be infused within our practice and our science, a critical mass of behavior analysts will need to deepen their study of it. Therefore, the purpose of this article is to support the design of behavioral instruction using DI principles and to prepare behavior analysts to take up the torch of DI. This article is intended to be a starting place, and to inspire new behavior analysts to learn further and to persuade experienced behavior analysts to give DI a try.

DI is often categorized according to program design, organization of instruction, and teacher–student interactions (Watkins & Slocum, 2004). However, for the purposes of this article, the DI principles and strategies have been organized into 10 critical instructional design efforts that can be completed in a strategic sequence and be used immediately. In addition, a one-page, easy-to-use Planning Guide is included in this article (Figure 1). As behavior analysts begin their first instructional design project, they can carry out the sequence of design efforts while referring to the guiding questions in the Planning Guide. To show that DI can be readily applied to behavior analytic practice with diverse individuals, several practical examples are integrated throughout. Finally, a more comprehensive illustration of the 10 design efforts—application of DI to teaching narrative language—is outlined in the Appendix. Readers interested in teaching complex academic language skills through stories are referred to Spencer and Petersen’s (2020) article on the principles of narrative intervention, which loosely align with DI.

**Fig. 1:**
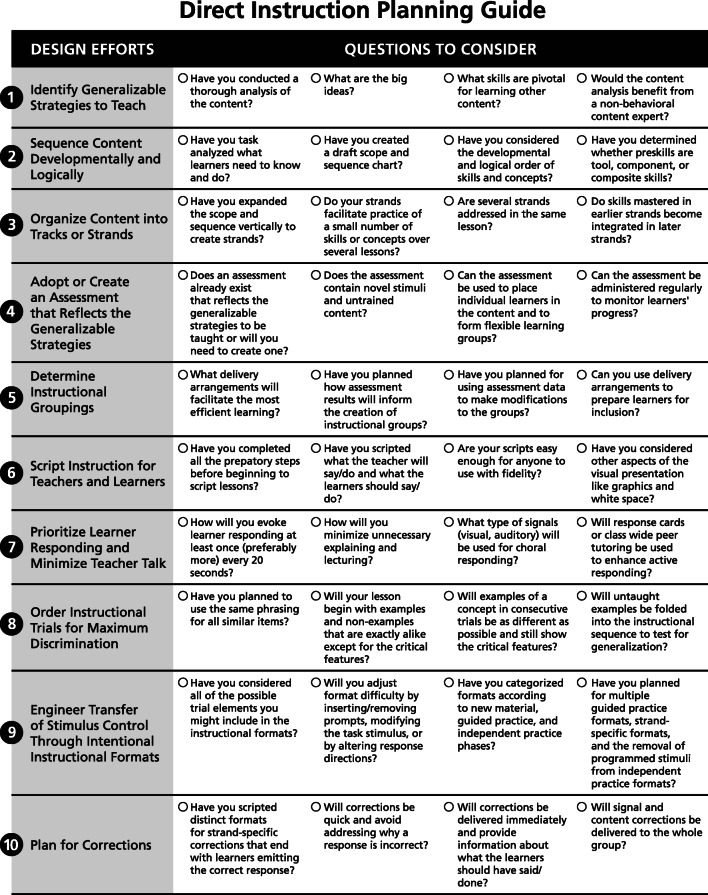
Direct Instruction Planning Guide

## Identify Generalizable Strategies to Teach

As you embark on an instructional design endeavor, the first thing to do is determine the broad, generative skills you will teach (Stein et al., 1998). This contrasts with traditional behavior analytic instruction which assumes such information emerges from assessment results of an individual learner (Ferraioli et al., 2005). A behavior analyst might review a child’s VB-MAPP results (Sundberg, 2014), identify several missing skills, and create programs tailored to the individual child’s barrier profile to teach those skills (Barnes et al., 2014). Although data certainly informs where a learner enters instruction, from a DI perspective, the design of instruction begins with an analysis of the content (Tiemann & Markle, 1990) and not an analysis of the learner. DI is best used for teaching the big ideas or generalizable strategies that all learners need to acquire. For example, rather than identifying that a learner does not yet point to truck, bus, and car according to the class *vehicles*, you determine that all learners will need (at some point in the future if not already acquired) to be able to point to, tact, and answer questions about *untrained* targets according to features, functions, and classes. The latter refers to skill repertoires that direct your attention from the learner to the content. Although this may be less comfortable for behavior analysts, designing instruction to teach the big ideas (i.e., generalizable strategies) will result in an instructional plan that can be used and reused with a variety of learners, saving valuable time. Please note that the current use of the term “strategy” contrasts from a common use of the word that means “tactic” or specific strategy a teacher might use. In this article, *strategy* refers to learners’ higher-level composite skills.

In DI, efficiency is key (Engelmann & Carnine, 1991). Efficient instruction is vital for clients with disabilities and/or learning differences. To achieve efficiency, the focus must be on teaching repertoires that facilitate and hasten the learning of other content (sometimes called pivotal skills or behavioral cusps; Smith et al., 2006). A common misunderstanding is that DI is used exclusively for teaching rote skills. Of course, mastery of basic skills is necessary (and can be taught using DI), but the purpose of mastering basic skills is to efficiently build complex generative repertoires, also called strategic or recombinative repertoires (Alessi, 1987). During an analysis of content, the instructional designer aims for the highest generativity ratio—the largest generative repertoire achievable in the least amount of instructional time (Slocum & Rolf, this issue). A generative repertoire, ensures that novel responses are possible without directly teaching every stimulus–response relation (Alessi, 1987). For example, after learning a set of 12 morphographs (*able, re, arm, claim, er, ing, cover, ed, dis, order, un, ness*), children should be able to combine them in various ways to form over 75 different words (Dixon, 1976). If you intend to teach generative repertoires to your clients, then DI can help.

Identifying the generalizable strategies (i.e., big ideas or target generative repertoires) requires a thorough analysis of the content to be taught. Behavior analysts’ training is on the principles of behavior and learning, not necessarily reading, math, science, social studies, second/foreign languages, social skills, vocational skills, etc. Unless you plan to teach the science of behavior, an analysis of content may require the involvement of a content expert. Behavior analysts should be able to exhibit sufficient professional humility to learn from colleagues whose training focuses on content such as reading and math teachers, vocational rehabilitation specialists, speech-language pathologists, and biologists. Their expertise can be critical for understanding the content sufficiently to design instruction.

It is worth mentioning here that DI applies to generalizable strategies of all shapes and sizes. Although it is easy to imagine a curriculum designed for a cluster of repertoires called reading, second language skills, social skills, or vocational skills, you may only be interested in a smaller segment of those repertoires. For example, a perfectly suitable (and yet extremely complex) repertoire of skills to which DI applies might be *social interaction skills necessary for activities in the community*. The critical feature of an acceptable target for instruction is that the strategy is indeed generalizable. In other words, is this cluster of skills needed in many contexts? Another critical feature is that the target strategy is comprised of several component skills, making it a teachable higher-level composite of skills and repertoires (Johnson & Street, 2012). *Social interaction skills necessary for activities in the community* encompass numerous component skills such as positioning oneself at an appropriate distance to cashier/server, looking at the cashier/server, making a clear and audible verbal request, and using “please” and “thank you,” to name a few.

## Sequence Content Developmentally and Logically

Once you have identified the generalizable strategies you plan to teach, the content needs to be sequenced (Tiemann & Markle, 1990). It takes time to do this well, but it will save time in the long run. Also, it is okay if you do not get the sequence perfect the first time. During this effort, it is helpful to create a scope and sequence chart (in a table or an excel file) with your generalizable strategies toward the end of the timeline. Working backwards, task analyze (i.e., break into smaller parts) each content segment to determine all the knowledge and skills that are required to achieve the subsequent objective. In this process, it is important to understand relevant preskills (i.e., prerequisites), the difficulty of each skill, and to identify skills that can be taught simultaneously. In this context, developmentally sequenced content reflects the necessary order of skills based on their prerequisites and difficulty. In other words, this refers to the development of the skill, not the development of a learner. Think logically about what the learner needs to *know and do* to be successful at each step within the content. It may be useful to watch skilled individuals engage in the target repertoires to ascertain the order in which the components occur. For example, observations of young adults making purchases at stores and restaurants can provide insight about the component social skills involved in these activities.

While breaking the content into smaller segments and sequencing them, consider labeling each prerequisite as a tool (basic), component (combines multiple tool skills), or composite skill (combines multiple component skills; Merbitz et al., 2004) so that you do not miss the nesting or hierarchical structure of skill building. If pointing to, tacting, and answering questions about untrained stimuli based on features, functions, and classes is the generalizable strategy, one necessary component skill is pointing to pictures of trucks after hearing the class “vehicles.” To perform this skill, however, several tool skills are needed such as reaching and pointing.

The purpose of this careful sequence of content is to prepare learners to be successful at each subsequent step and to maintain the momentum necessary for increasingly difficult tasks (Nevin & Grace, 2000). Logic demands that prerequisite skills be taught before the strategy that depends on them (Watkins & Slocum, 2004). Engelmann (2007) used the analogy of a staircase to describe how each step should be just the right size, not too large and not too small. If learners are well-prepared, they will be less likely to make errors as they traverse the metaphorical staircase. Fewer errors hastens the acquisition of new knowledge and skills, thereby contributing to instructional efficiency.

## Organize Content into Tracks or Strands

After producing a draft scope and sequence of the content, you may discover that is not as linear as you would like it to be. In reality, there are many skills that can be addressed simultaneously and many preskills can be taught in any order. Although it adds a layer of complexity to instructional design, a vertical expansion of your scope and sequence chart will facilitate the creation of tracks or strands, which is somewhat unique to DI (Snider, 2004). Tracks (or strands) are series of activities that teach the same skill/concept across many lessons. For example, teaching item-class relations (e.g., categorization track) would be distributed across several weeks of instruction, while ensuring multiple classes and many items (and multiple exemplars of items) within each class are included in the instruction. In addition, each lesson contains activities for several tracks (Watkins & Slocum, 2004). Imagine a lesson that contains three activities, one for each of the three tracks it addresses: (1) tacting unknown items and new exemplars of recently introduced items, (2) pointing to an item based on function, and (3) sorting items by class. This type of instruction is significantly different from traditional instruction, which is often organized by themes or topics that spiral every year. Meaning, instruction and practice of content only takes place during the unit and once the unit is over, students no longer use the knowledge and skills acquired. Spiral instruction impedes retention of material and increases the need for reteaching, which is extremely inefficient (Engelmann, 2007; Snider, 2004).

**Fig. 2:**
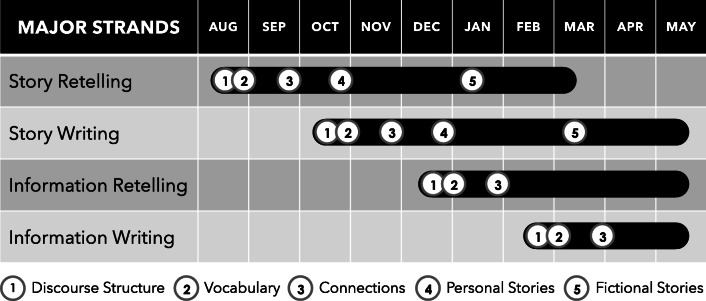
Example of a Scope and Sequence Chart for Teaching Academic Language

The strand design of DI enables regular and steady practice of a small number of concepts or skills over a longer period of time (see Figure 2 for an example of strands related to academic language). As learners become proficient, tool or component skills are integrated with other tool and component skills to build composite skills. Once a strand is mastered, the strand skills and concepts are used to learn the content in a new strand (Engelmann, 2007), creating an increasingly complex and interwoven network of content until eventually the generalizable strategies are mastered. In the example of pointing to, tacting, and answering questions about untrained stimuli based on feature, function, and class, one strand could represent *features*, another *functions*, and another *classes*. If there is not a developmental or logical order to the feature/function/class strands, all three can be addressed within the scope and sequence simultaneously (but do not have to be). However, when there is a developmental or logical order, content should be taught in that order. It may be necessary to begin teaching *pointing to based on feature/function/class* before *tacting based on feature/function/class* and to teach *tacting based on feature/function/class* before *answering questions based on feature/function/class*. Instructional strands that precede *feature/function/class* strands might include *pointing to* and *tacting pictures/objects*. Once mastered, these basic skills become interwoven with *feature/function/class* strands.

Strand construction facilitates mastery and maintenance because the practice is distributed over time and then strategically applied in later lessons. However, strands also aid in differentiation. There will be learners who require reteaching or who present with a smattering of skill deficits unrelated to their overall abilities. A detailed scope and sequence chart with strands will help you determine an entry point or a temporary backtrack for an individual learner with weak, repertoire-necessary skills. For example, if when teaching purchasing skills, you notice that your learner is not using an appropriate volume of speech, which may be a skill that should have been mastered earlier, you can use the scope and sequence chart to help plan which lessons need to be repeated.

## Adopt or Create an Assessment that Reflects the Generalizable Strategies

For behavior analysts, the link between instruction and assessment is easily understood. However, the type of assessment needed for DI may be less familiar to some. In DI, assessment fulfills several purposes, including placing individuals in the content sequence, grouping learners, modifying instruction, and determining mastery (Watkins & Slocum, 2004). These purposes are achieved without capturing response data on every instructional trial or measuring the mastery of taught targets. With DI, it is customary to use curriculum-based measurement (reflects the content, but is not the exact content taught) to identify where learners’ repertoires fall in the sequence of lessons, but thereafter on a regular basis (e.g., weekly, biweekly), monitor learners’ progress toward achievement of the generalizable strategies (Deno, 2003). This type of assessment uses novel stimuli to examine learners’ ability to generalize from trained to untrained material, which is necessary when teaching generalizable strategies. Because the instructional and assessment content reflects generalizable strategies, it does not make sense to document performance on each opportunity to respond during instruction.

Depending on the generalizable strategies addressed in your instruction, you might be able to adopt an assessment tool that already exists. If relevant assessment instruments are not commonly used among behavior analysts, then search for usable tools in other disciplines. For example, oral reading fluency (Fuchs et al., 2001), borrowed from education, is standardly measured through curriculum-based measurement. Each time a learner’s reading fluency is measured, a different passage is used. Some speech-language pathologists collect regular language samples using a variety of wordless picture books (Miller et al., 2016) and examine the samples for complex sentences (e.g., subordinate clauses). In each of these examples, assessment data can be graphed across time and progress is shown in the number of words read correctly per minute or number of subordinate clauses per 100 words spoken, respectively. If an assessment that captures the generalizable strategies you plan to teach does not exist, you may have to create one. Keep in mind the decisions that need to be made (e.g., placement in content, forming groups, modifying instruction, and determining mastery of generalizable strategies) and that assessment should not be conducted using the same materials and specific examples targeted during instruction. As you use the assessment tool, you will identify ways to increase its utility, feasibility and reliability. Iterative improvements are expected.

Imagine you need to create an assessment tool that reflects the generalizable strategy of pointing to, tacting, and answering questions about untrained stimuli based on features, functions, and classes. The assessment would need to include items/questions that reflect each response form (i.e., pointing, tacting, and answering) as well as items/questions that reflect each discrimination (i.e., features, functions, and classes). Sequence the items/questions from easy to difficult so that you can discontinue the assessment if necessary and still obtain a graphable score. As instruction continues, learners will likely respond correctly to more items. The selection of the stimuli and items/questions to include in the assessment is the most crucial part. Novel stimuli of trained targets (e.g., pickup truck, bus, and sedan car) and untrained targets (e.g., semi-truck, helicopter, SUV) are needed so that both stimulus generalization and concept acquisition can be assessed. Once an assessment template is established, multiple forms of the assessment need to be created so that novel stimuli and untrained targets can be substituted for repeated monitoring of the generalizable strategy. It is important to plan for novel stimuli and novel items/questions for the assessments as the scope and sequence is finalized, ensuring that a set of stimuli/targets is reserved for assessment of the final generalizable strategy.

It is not necessary to restrict assessment to examiner-delivered items; the critical features of an appropriate assessment tool might be present in the natural environment. For example, if you want to assess *social interaction skills necessary for activities in the community*, you would only need to plan for novel activities and settings within the community in which the social interaction skills are observed. Opportunities for all social interaction components would need to be present in every assessment observation and the assessment contexts would need to be different than the contexts used for training. Finally, use the same measurement and graphing system for each observation so that improvements over time can be detected.

## Determine Instructional Groupings

Before writing lesson plans, you need to determine the arrangement(s) for instructional delivery. For example, will instruction be delivered to large groups of learners, small groups, or individually? If the answer is all types of arrangements, then the language used in your lessons must be broadly applicable and the manner in which opportunities to respond are engineered should be considered carefully. The other option is to develop versions of the same lessons that are tailored to various group sizes. One-on-one instruction may be more common in behavior analytic practice, but it is less efficient than group instruction. Typical behavior analytic instructional environments (e.g., clinics, homes, centers) are often not the learners’ natural educational settings (e.g., mainstream general education classroom). While planning your content and instruction, strive to understand your population’s target learning environments and typical delivery arrangements so that your lessons can prepare learners for later learning and inclusive experiences. Instruction that begins with a one-on-one arrangement, but progresses to small group, and then to large group arrangements may help learners transition to typical learning environments better than one-on-one instruction alone. Familiar instructional characteristics can ease exceptional learners gradually toward increased demands. DI is an excellent tool for preparing learners with disabilities for inclusion because the design principles and content remain the same across all delivery arrangements (Watkins et al., 2011).

It is possible that some elements of your scope and sequence need to be delivered in a one-on-one arrangement. For example, it could be very challenging to teach *pointing to pictures based on features/functions/classes* in a small or large group because materials are needed. Also, given its motoric response, learners can easily imitate others in the group, which may be counterproductive. Of course, there are several clever methods for teaching listener behaviors in groups and sometimes the objective is for learners to respond to directions given to a group. In this example, however, it would be feasible to begin small group instruction for *tacting and answering questions based on features/functions/classes* once *pointing to pictures based on features/functions/classes* is mastered. All of these teaching objectives can still be included in the same scope and sequence plan.

Flexible learning groups is a hallmark DI strategy that contributes to the efficiency of instruction. DI is most often used in classrooms led by one or two teachers, and more can be accomplished in less time in group instruction. Flexible grouping means that learners can be regrouped at any time, depending on their performance or rate of performance evidenced by progress monitoring results. Learners should begin instruction where they can be successful based on individual achievement of prerequisite skills and be grouped with other learners who are similarly prepared. Once learners are initially placed into the curriculum and organized into groups according to the screening assessment (also called placement tests), be prepared for learners to progress differently (Watkins & Slocum, 2004). Progress monitoring data are used to make changes to groups in a fluid manner. For example, a learner may be struggling with a specific concept and need to be placed in a different group that is just beginning the strand related to that concept. Likewise, one learner may be advancing quickly and become bored as the other learners go at a slower pace. They can also be regrouped to ensure more homogeneous groupings.

## Script Instruction for Teachers and Learners

As the design efforts transition to the creation of the lessons themselves, an appreciation of the preparation that precedes this step is warranted. It is a mistake to skip the preparatory efforts that require the instructional designer to think carefully about what they plan to teach, the order in which it should be taught, and the relation of the content to assessment and instructional groupings. However, those who have thought carefully about the content will be ready to craft the lessons. From a DI perspective, lessons are detailed plans for teacher–student interactions, not general statements about what is going to be accomplished during the lesson, a list of materials to use, or suggested teaching tactics. DI lessons are explicit “implementation supports” (Parnell et al., 2017; Sanetti & Collier-Meek, 2015) teachers rely on to deliver the instruction. They are scripted to promote efficient, high fidelity delivery.

Scripted instruction is familiar to behavior analysts who used discrete trial teaching. For a specific discrete trial program, there is usually a preplanned antecedent direction, a description of correct behavior(s), and a specified consequence procedure (Smith, 2001). It is up to the clinician delivering the trials to engineer the order and frequency of specific targets, stimuli to be used for each trial, schedules of reinforcement, fading of prompts, etc. Although many clinicians can do this well, it requires a great deal of competency-based training and supervision, and most teachers and behavior analytic technicians do not have sufficient training and experience to avoid mistakes (Clayton & Headley, 2019; Parnell et al., 2017).

**Fig. 3:**
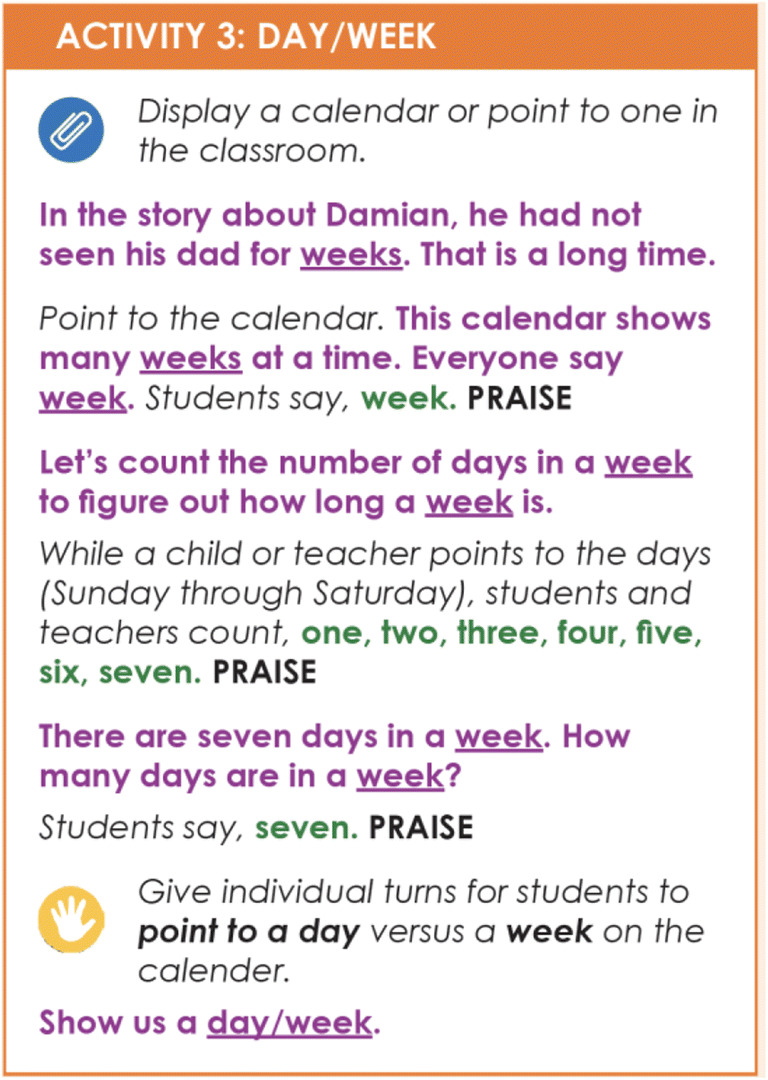
Example of Scripted Lesson Activity

DI scripts make it possible to deliver sophisticated instruction across mixed operants (i.e., multiple strands), varied targets, and a range of materials. Scripts allow for preplanned and precise alterations in discriminative stimuli and prompts so that systematic shaping of independent responses is largely inevitable. The elaborated scripts used in DI lessons, describe in dialogue, every teacher-student interaction needed for 5–30 min of instruction and intentionally integrates the practice of several strands as it builds learners’ skills toward generativity (Engelmann, 2007; Snider, 2004). Figure 3 shows an example of scripted dialogue for teaching how many days there are in a week to preschoolers. Because the number of instructional decisions necessary for weaving the strands within and across lessons is so high, they cannot be made on the fly, even by well-trained, experienced clinicians or teachers (Hummel et al., 2004; Watkins & Slocum, 2004). Sophisticated instructional design requires considerable thought and preparation; writing it down is the best way to ensure it can be delivered effectively. DI is efficient and potent, largely because scripted DI lessons can be delivered with few mistakes.

## Prioritize Learner Responding and Minimize Teacher Talk

Learners must behave to contact the consequences that determine whether they will engage in that behavior again. This instructional design effort involves the creation of teacher–student interactions that promote a brisk pace, frequent opportunities for learners to respond, and judicial use of time, all of which are designed to ensure learners sufficiently contact relevant reinforcers. When developing scripted lesson plans the attention needs to be on what the learners will do and what the teacher will do to evoke correct responding (Engelmann & Carnine, 1991). Instructional sessions are not the time for lengthy explanations or lecturing, but for modeling and reinforcing approximations of target skills. The focus on learners’ active responding helps to maximize the instructional time, which yields efficiency (Ellis & Worthington, 1994). Maintaining a brisk pace of trial delivery that results in frequent correct responding reduces behavior problems and increases on-task behavior, while accelerating learning (Archer & Hughes, 2011; Forsyth & Archer, 1997; Greenwood et al., 1984; Tincani et al., 2005). For some learners, efficiency can be achieved through one-on-one instruction, but it is a mistake to assume that learners with disabilities cannot learn successfully in groups. Small or large group instruction that prioritizes learner responding over explaining and lecturing can be effective for learners with significant disabilities (Kamps et al., 2016; Plavnick & Dueñas, 2018; Thompson et al., 2019).

High numbers of opportunities to respond can be achieved in group instruction. In DI, choral responding is the primary strategy to achieve this. When responding chorally, the group provides an oral answer together (Heward & Wood, 2015). Teachers deliver a distinct signal (e.g., hand drop, snap, tap, clap) to cue the timing of unison responding much like a choir director motions for a choir to sing (Watkins & Slocum, 2004). As you develop your lessons, do not forget to include guidance and scripts to facilitate group responding (e.g., “Everyone, _______.”) and consider the form of signal that will work best. For example, visual signals (e.g., hand gestures) work well if the learners are able to look at the teacher, but if learners need to attend to stimuli in a book or on a table, then an auditory signal will be needed (e.g., snap or tap). Choral responding ensures all learners receive high quality instruction because each one produces their own response, and because responding is timed with a signal, they cannot imitate other learners. Furthermore, when learners respond frequently, teachers can assess each learner’s skills within the lesson informally and make on-the-spot instructional decisions contingent upon responding.

**Fig. 4:**
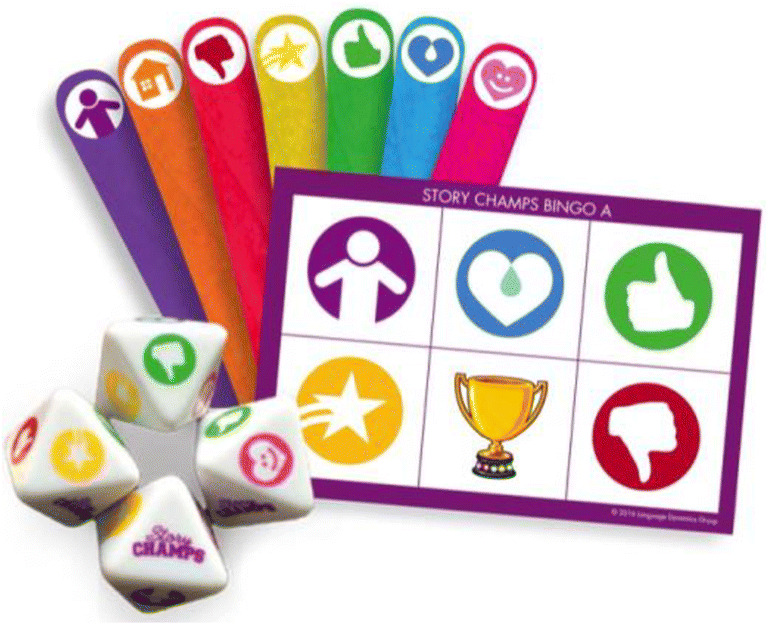
Examples of Response Card Systems

For responding that is not oral, response cards (Heward et al., 1996; Schnoor et al., 2016) are another way to accomplished precisely timed group responding. Response cards are notecards, signs, small white boards, etc. that students display so the teacher can see to indicate a silent response. They can be preprinted or written just prior to the signal indicating it is time to show the teacher. Response card methods are seen in several educational technologies available today (e.g., Clickers, Kahoot!; see Twyman & Heward, 2018) and may take many forms (see Figure 4 for response cards made for listening to stories). Regardless of the method used to facilitate active responding, your lessons should be crafted so that all your learners respond at least once (preferably more) every 20 s (MacSuga-Gage & Simonsen, 2015).

## Order Instructional Trials for Maximum Discrimination

Presenting examples and nonexamples in a preplanned order to highlight critical similarities and differences in DI lessons is called *exemplification* (Engelmann & Carnine, 1991). This instructional design effort will lead to lesson plans that help even the novice of teachers conduct expert discrimination training. It is important to note that the selection and juxtaposition of examples and nonexamples are vital to accomplish the general case programming needed for DI (Engelmann & Becker, 1978). Generative responding culminates from a set of strategically sequenced exemplars (and nonexemplars) of a concept. If the goal is for learners to respond correctly to novel stimuli that represent the concept, you must avoid teaching discrete cases. This can be accomplished using what Engelmann and Carnine (1991) call *faultless communication*. In other words, the arrangement of examples and nonexamples is so clear and so elegant that the concept’s distinguishing characteristics are readily apparent (Twyman, 2021). If done well, learners abstract the critical features of a concept from the examples and are not distracted by the irrelevant features.

Johnson and Bulla (this issue) and Twyman (this issue) provide thorough reviews of how to select and order examples and nonexamples to train crucial discriminations. However, here a few simple rules are presented that can guide the ordering of instructional trials within a lesson (Engelmann & Carnine, 1991; Watkins & Slocum, 2004). First, use the same phrasing for all similar items. When only the critical feature(s) of the instruction vary (e.g., “A bus is a vehicle. A bowl is *not* a vehicle.”), the differences are easily detectable. More than one instructional variation at a time confuses learners because they have to determine what is relevant and what is not. Second, begin the lesson with examples and nonexamples that are exactly alike except for the critical distinguishing feature (e.g., a yellow school bus and a yellow shed with a curved roof). As exemplification continues within and across lessons, the examples and nonexamples can differ by more than one feature (e.g., a city bus and a metal gate). Third, examples of a concept in consecutive trials should vary as much as possible while still illustrating the same critical feature(s) (e.g., example of boat followed by example of helicopter). Examples (e.g., truck) and nonexamples (e.g., plate) can be repeated within a lesson, but careful attention should be given to show multiple exemplars of each example (e.g., several different types of trucks) and nonexamples (e.g., several different types of plates). Fourth, untaught examples should be folded into the sequence of instructional trials to test for concept acquisition. In other words, when shown a novel example of the concept, do learners recognize it? Learners’ performance on intermittent opportunities to apply new knowledge and skills reveals the extent to which generativity is achieved, also known as concept acquisition.

The guidelines described above can be applied easily to much of what behavior analysts teach their clients, but for some content, the application is more challenging. When the content is difficult to depict with objects or pictures (e.g., *social skills*, *storytelling, vocational skills*), it is important to remember the purpose of exemplification, in general. Regardless of the format of examples and whether nonexamples can and should be included, their selection and introduction should be strategically designed to achieve efficient discrimination of concepts.

## Engineer Transfer of Stimulus Control through Intentional Instructional Formats

It can be argued that programming for the transfer of stimulus control is the most important aspect of effective instruction. This is best accomplished with the use of instructional formats that systematically vary the difficulty of trials and independence of targeted learner responses. Instructional formats are general sets of trial elements, much like manipulative autoclitic frames (Alessi, 1987), that are arranged in a strategic order within and across lessons. Familiar trial elements include antecedents, responses, and consequences, but DI instructional designers regularly make use of additional trial elements such as attention signals, task stimuli, stimulus directions, stimulus prompts, response prompts, response directions (i.e., antecedents or discriminative stimuli), and signals (Becker & Engelmann, 1975; Kame’enui & Simmons, 1990; see Table 1 for all possible trial elements). To configure instructional trials for the transfer within and across lessons, multiple instructional formats (i.e., multiple general sets of trial elements) are needed. It is through the intentional addition, modification, or removal of trial elements within instructional formats that learners’ independence is increased while programmed prompts are faded.

**Table 1:** Expanded Instructional Trial with Possible Format Elements

Possible Format Elements	Descriptions of Format Elements	Examples
Attention Signal	Request for learners' attention	"Everyone, watch me."
Task Stimuli	Materials needed for the trial	Picture of a bus
Stimulus Direction	Request for learners to look at stimuli	"Look at this picture."
Stimulus Prompt	Draw attention to key features of stimuli	"Buses have tires," while pointing to the tires.
Response Prompt	Model of the response or approximation of the response expected of learners	"A bus is a vehicle."
Response Direction	Discriminative stimulus that communicates to the learner what response will be reinforced	"What is a bus?"
Signal	Visual or auditory cue for when learners should respond	Finger snap
Response	What the learner says or does	"A vehicle"
Feedback	Reinforcement or correction	"Yes, a bus is a vehicle."

Response direction, response, and feedback are the only obligatory elements of an instructional trial/format and there are three key methods for adjusting the difficulty of trials, which are needed to create multiple instructional formats. First, the insertion of stimulus or response prompts makes a trial easier and their removal makes it more challenging. Response prompts are typically the most direct form of prompting, but their magnitude and topography can be modified to be more or less helpful to the learner. For example, a response prompt that models 100% of the target response (e.g., “vehicle”) makes the trial easier for the learner than a response prompt that only approximates the target response (e.g., “vvv”). Stimulus directions and stimulus prompts are not always needed, but can be subtle forms of support. If the response direction was “How does this boy feel?” a stimulus direction “Look at his face” or a stimulus prompt “He is smiling” should increase the probability of the learner responding correctly.

The second method for modifying an instructional trial is to manipulate the task stimulus itself (i.e., materials). Stimulus fading, positioning stimuli, or highlighting relevant features of a stimulus are examples. The number, array, and types of stimuli selected (e.g., photos or illustrations) are also relevant to trial difficulty and the deliberate transfer of stimulus control.

Varying the response direction is the third method of modifying trial difficulty. Learners’ responses are directly linked to response directions so altering the phrasing of a response direction can change the expected response form. For instance, in a listener task, the response direction might be “Point to the vehicle” and in a tact by class task, the response direction might be “What is a bus?” A subtle alteration of the latter response direction might be “Is a bus a vehicle or clothing?” The choice-of-two response direction would be included in formats that occur earlier in the lesson than “What is a bus?” but later than “Point to the vehicle.”

**Fig. 5:**
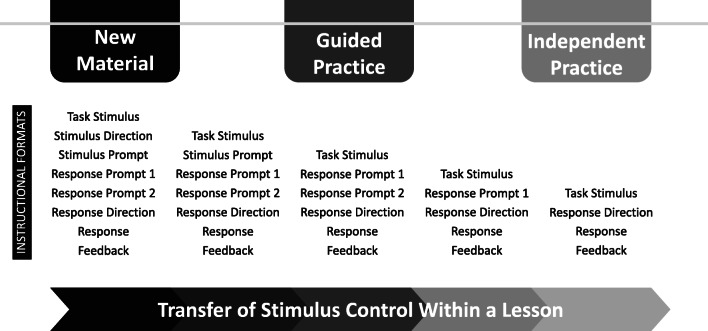
Example Instructional Formats Across Teaching Functions

When creating instructional formats for your lessons, it is helpful to categorize them according to new material, guided practice, or independent practice (see Figure 5 as an example of instructional formats across the teaching functions). According to these teaching functions (Archer & Hughes, 2011; Hofmeister & Lubke, 1990), the formats used when introducing new content should contain more supports and evoke easier approximations of the target behavior and formats during independent practice should be absent of supports and evoke target responses. Guided practice is the phase between new material and independent practice that facilitates the transfer (Kame’enui & Simmons, 1990).

Although many instructional formats are possible, a common DI format for teaching new material is to include two types of response prompts, the purpose of which is to prevent errors. This involves a model of the expected response (e.g., “A bus is a vehicle.”) followed by a lead (e.g., “Say it with me. A bus is a vehicle.”), which requires the learners to repeat the model as the teacher says it. Immediately following the lead, the response direction is delivered (e.g., “What is a bus?”) to evoke an independent response (e.g., “A vehicle.”). Notice how the second response prompt (i.e., lead) shares features with both the first response prompt and the response, forming a smooth bridge from the model to an independent learner response. This promotes a speedy transfer of stimulus control from programmed instructional stimuli (e.g., prompts) to the natural antecedent (e.g., question), and allows learners to contact reinforcement for independent responses quickly. In later presentations of the same content, in guided practice for instance, the lead step can be dropped. The content eventually progresses to the independent practice phase in which prompts are removed. It becomes more efficient at that point to rely on correction procedures if an occasional error occurs.

There are five additional considerations to keep in mind about instructional formats. First, you can use several instructional formats during guided practice, if necessary. The bulk of each lesson should be spent in guided practice so depending on your content, you might need to withdraw or modify trial elements more slowly, which would result in more than three instructional formats within the lesson (see Figure 5).

Second, the design of instructional formats within a lesson should be content/strand specific. For example, teaching vocabulary requires different types of instructional formats than teaching discourse structures. Likewise, teaching listener behaviors necessitates a different set of instructional formats than teaching tacting skills.

Third, every time you introduce a new exemplar for instruction (e.g., ordering food at restaurant, purchasing clothes at a store, shopping for groceries) within a strand, you need to start with the instructional format for new material. Each exemplar needs to go through the teaching functions (i.e., new material, guided practice, and independent practice) independently; however, as instruction continues the amount of time it takes to go through the different teaching functions for subsequent exemplars may decrease. Random or periodic tests for concept acquisition are the exception—in other words, inserting a test for generalization (using an untrained exemplar) necessitates trials that look like independent practice trials.

Fourth, it is reasonable to have some exemplars in new material whereas other exemplars are in guided or independent practice within the same lesson. When only 20% of lesson content addresses new material, learners remain motivated (Hofmeister & Lubke, 1990). Depending on the content, a single lesson could teach *Bus* as new material and provide guided practice for *Truck* if it had been introduced in a previous lesson. This also applies to strands. When teaching academic language, discourse structures are often in guided or independent practice phases when complex sentences or vocabulary are introduced.

Finally, it is imperative that instructional designers remove all the programmed stimuli from the final instructional formats so that unintentional stimuli do not impede the transfer of stimulus control (e.g., young adult enters a store to make a purchase without a teacher shadowing). If this is not attainable within every lesson, the final instructional formats can be shaped across a series of lessons so that they eventually yield the purest trials possible (i.e., only the natural antecedents are present). For example, if at the end of the first five lessons, it is unreasonable for learners to respond independently without any form of prompt, then the prompts in independent practice instructional formats should be faded gradually in consecutive lessons.

## Plan for Immediate Corrections

Although DI relies heavily on the strategic arrangement of antecedents, effective instructional designers do not leave consequences to chance. In fact, the work to engineer instructional antecedents carefully is in spirit of promoting quick and consistent contact with reinforcement. When learners do not contact reinforcement (e.g., emit an incorrect response), additional teaching is conducted in the form of a correction. In DI, corrections are extra instructional formats delivered contingent upon errors. In many instances, they look a lot like the instructional formats designed to prevent errors. For example, DI designers often use two types of standard correction formats: model-lead-retry and model-retry. Either of these works well for discrete skills such as defining a vocabulary word (e.g., “Grimy means very dirty. Say very dirty with me. Very dirty. What does grimy mean?” or “Very dirty. What does grimy mean?”), but model-lead-retry provides more support than model-retry. Notice, in these standard corrections, that time is not spent talking about why an error was made or what was wrong with the response. Effective corrections provide immediate feedback on what learners should have said/done and always end with the learners emitting the correct response (Archer & Hughes, 2011; Watkins & Slocum, 2004). Keep in mind corrections can be physical in nature. For example, a lead step may look much like full physical prompting or graduated guidance.

When the response direction is delivered to a group, corrections are also delivered to the group (Watkins & Slocum, 2004). Children who receive individual attention for their errors may be less likely to try difficult things. Group error corrections also give the whole group a little more practice and provide informal assessment information to the teacher. Although to this point content corrections have been the focus, teachers should also be prepared to correct signal errors. When a group of learners does not respond on signal in unison, whether or not the content of the response was correct, the signal should be corrected (e.g., “I need to hear everyone together on my signal. Let’s try it again. What does grimy mean?”). If signal errors are not corrected, they will slow the pace of instruction and allow weaker learners to imitate others.

## Call to Action

Behavior analysts have mastered discrete trial instruction, yet few are prepared to take advantage of DI, another instructional approach that would likely benefit their clients. DI is a specialized form of instruction in which efficiency and potency are paramount. The power of DI is seen when leaners become better at learning. In this article, 10 critical instructional design efforts were introduced and several examples illustrated the flexible application of DI to behavior analytic practice. These design efforts can be applied to a large array of skill repertoires and with diverse learners. DI is a worthy endeavor for behavior analysts; more important, it is easy to get started using the Planning Guide. Although it is not meant to be comprehensive, the Planning Guide should be a sufficient reminder of the principles and guidelines covered in this article. This article was designed to help get you started using DI, but the coverage of the theory of instruction was not exhaustive. The other articles in this issue should be studied thoroughly as the authors have covered some of the most important DI design efforts in more depth (and ambitious learners should read Engelmann & Carnine, 1991). Skillful instructional design requires time and practice, but the efficiency, flexibility, and utility of DI are worth it. It is time for behavior analysts to take up the torch Engelmann lit and left burning for us. It is now our responsibility to make sure the fire does not go out and that it spreads liberally within and beyond behavior analysis.
